# [^18^F]NaF PET/CT Imaging of Iliac Bones to Assess Bone Turnover

**DOI:** 10.1007/s11307-025-02003-6

**Published:** 2025-04-24

**Authors:** Shashi B. Singh, Om H. Gandhi, Bimash B. Shrestha, Patrick Glennan, Anuradha Rosario Bahadur, Niloofaralsadat Motamedi, Kishor Khanal, Sagar Wagle, Poul Flemming Høilund-Carlsen, Thomas J. Werner, Mona-Elisabeth Revheim, Abass Alavi

**Affiliations:** 1 Hospital of the University of Pennsylvania, 3400 Spruce St, Philadelphia, PA 19104 USA; 2 Kingston Public Hospital, North Street, Kingston 4, Jamaica; 3 Department of Cardiology, University of Arizona, 1200 E University Blvd, Tucson, AZ 85721 USA; 4 Enloe Medical Center, Chico, CA 95926 USA; 5 Department of Nuclear Medicine, Odense University Hospital, Odense, Denmark; 6 Department of Clinical Research, University of Southern Denmark, Odense, Denmark; 7 The Intervention Center, Division for Technology and Innovation, Oslo University Hospital, Nydalen, Post box 4950, 0424 Oslo, Norway; 8 Institute of Clinical Medicine, Faculty of Medicine, University of Oslo, Problemveien, 0313 Oslo, Norway

**Keywords:** Iliac bone, [^18^F]-sodium fluoride ([^18^F]NaF), Positron emission tomography (PET), Positron emission tomography/computed tomography (PET/CT), Osteoporosis, Bone, Metabolism

## Abstract

**Purpose:**

This study investigated the effects of laterality, age, gender, BMI, and physical activity level on iliac bone turnover using [^18^F]NaF PET/CT.

**Procedures:**

Fifty-nine males and 44 females from the CAMONA study were analyzed. A region of interest (ROI) was drawn to segment the iliac bone using Hounsfield unit thresholds and morphological closing algorithm. [^18^F]NaF SUVmean was compared between the left and right iliac bones using a paired t-test, while Pearson correlation coefficient assessed changes with age, BMI, and physical activity level.

**Results:**

[^18^F]NaF uptake was higher in right iliac bone than left in males, females, and the combined-group. In males, SUVmean was 2.98 ± 1.63 (1.1–7.87) on left and 3.71 ± 1.49 (1.49–3.7) on right. In females, SUVmean was 2.59 ± 1.14 (0.88–6.27) on left and 3.72 ± 1.04 (2.22–6.51) on right. Combined, SUVmean was 2.81 ± 1.44 (0.88–7.87) on left and 3.71 ± 1.31 (0.89–8.07) on right. [^18^F]NaF uptake negatively correlated with age (right: r = - 0.27, *P* = 0.006; left: r = - 0.22, *P* = 0.02), stronger in females (right: r = - 0.30, *P* = 0.04; left: r = - 0.31, *P* = 0.04) than males (right: r = - 0.26, *P* = 0.04; left: r = - 0.18, *P* = 0.18). SUVmean correlated positively with BMI in males (right: r = 0.47, *P* = 0.0002; left: r = 0.38, *P* = 0.0027), females (right: r = 0.36, *P* = 0.0168; left: r = 0.30, *P* = 0.0505), and combined-group (right: r = 0.43, *P* < 0.0001; left: r = 0.37, *P* = 0.0001). No significant correlation was found between SUVmean and physical activity in males, while in females, a negative correlation was observed on left (r = - 0.37, *P* = 0.0390) but not on right (r = - 0.27, *P* = 0.1302), and when combined, the correlation remained significant on left (r = - 0.24, *P* = 0.0372) but not on right (r = - 0.16, *P* = 0.1541).

**Conclusions:**

[^18^F]NaF uptake was higher in the right iliac bone and declined with age, particularly in females. The positive correlation between SUVmean and BMI; and the negative correlation between SUVmean and physical activity suggest metabolic influences on bone turnover. [^18^F]NaF PET/CT may serve as a tool for assessing bone metabolism and turnover in asymptomatic individuals.

## Introduction

Osteoporosis, a metabolic bone disease characterized by compromised bone strength and increased skeletal fragility, poses a significant public health burden due to its rising prevalence and consequences of osteoporotic fractures. As reported by the National Health and Nutrition Examination Survey, the overall age-adjusted prevalence among American adults aged 50 and over increased substantially from 9.4% in 2007–2008 to 12.6% in 2017–2018, with a disproportionately higher rise in females (14.0% to 19.6%) compared to males (3.7% to 4.4%) during this period [1]. The increasing incidence of fragility fractures, particularly involving the vertebrae, hip, distal radius, proximal humerus, and pelvis, is a major cause of morbidity, disability, and mortality in this population [1, 2]. Consequently, early and accurate diagnosis is crucial for the timely initiation of therapeutic interventions to mitigate further bone loss and reduce fracture risk.

Current clinical guidelines by the International Society for Clinical Densitometry recommend the use of dual-energy X-ray absorptiometry (DXA) to measure areal bone mineral density (BMD) at the lumbar spine, hip, or distal one-third of the radius for assessing fracture risk [3–5]. However, DXA, as the standard imaging modality for osteoporosis, has several inherent limitations. As a two-dimensional projection technique, it cannot discern the structural properties of bone nor differentiate between cortical and trabecular compartments [6]. Moreover, DXA measurements are susceptible to artifacts from degenerative changes, overlying calcifications, misplaced regions of interest, and improper positioning [7]. Importantly, while low BMD confers an increased relative risk of fracture, there exists a substantial overlap in the absolute risk among individuals with and without osteoporosis by DXA criteria [8]. In fact, most fragility fractures occur in patients who do not meet the BMD threshold for osteoporosis, likely attributable to the inability of DXA to capture bone quality characteristics that contribute to skeletal strength beyond density [9]. Bone histomorphometry involving a trans-iliac crest biopsy can provide static and dynamic indices of bone remodeling at the tissue level but is seldom employed clinically due to its invasive nature [10]. From a pathophysiologic perspective, the molecular derangements and dysregulation of bone cell activities that initiate osteoporosis precede detectable deficits in bone mass on structural imaging modalities like DXA for years or even decades [11]. As such, functional nuclear medicine techniques capable of capturing these early cellular aberrations hold significant promise for earlier detection of disease.

[^18^F]-sodium fluoride ([^18^F]NaF) positron emission tomography/computed tomography (PET/CT) imaging has emerged as a powerful molecular tool for the assessment of bone metabolic activity and remodeling, extending beyond mere density evaluations [12]. [^18^F]NaF is a bone-specific radiotracer that preferentially accumulates in skeletal regions with active mineralization fronts according to blood flow and remodeling space [13]. The degree of radiotracer uptake reflects the overall skeletal metabolic activity and remodeling status, with relative decreases indicating excessive bone resorption relative to formation, as seen in osteoporosis [14]. [^18^F]NaF PET/CT has been shown to improve the detection of osteoporotic vertebral fractures compared to conventional bone scintigraphy and volumetric bone mineral density assessment [15]. Prior investigations have leveraged this functional imaging approach to evaluate osteoporosis-related changes in bone turnover and density at axial skeletal sites like the spine and proximal femur [16, 17]. However, the utility of [^18^F]NaF PET/CT for assessing changes in bone metabolism of the pelvic bones has been relatively unexplored. Given the central load-bearing role of the pelvis and established susceptibility to fragility fractures, the iliac bone represents an intriguing site to investigate molecular signatures of systemic osteoporosis using [^18^F]NaF PET/CT. This retrospective study aims to evaluate the effects of laterality, age, gender, BMI, and physical activity level on iliac bone metabolism using [^18^F]NaF PET/CT.

## Materials and Methods

### Subject Selection

Participants in this retrospective study were identified from the prospective Cardiovascular Molecular Calcification Assessed by [^18^F]NaF PET/CT (CAMONA) study, conducted in 2012 at Odense University Hospital in Odense, Denmark. The CAMONA study was conducted in alignment with the principles outlined in the Declaration of Helsinki, with all participants providing written informed consent. The study received approval from the Danish National Committee on Biomedical Research Ethics and was registered at ClinicalTrials.gov (NCT01724749). Eligibility for inclusion as a healthy subject in the CAMONA study required an absence of any history of immunodeficiency, autoimmune diseases, cardiovascular disease, malignancy, pregnancy, drug abuse, or mental illness. The study, however, did not collect information on skeletal pain, previous trauma, or other skeletal issues. Each subject provided written informed consent.

### Image Acquisition

The study used harmonized PET/CT scanners (GE Discovery 690, VCT, RX, and STE) at Odense University Hospital to scan the subjects. PET images were taken 90 min after administering 2.2 MBq of [^18^F]NaF per kilogram of body weight intravenously, and the duration of emission acquisition was 2.5 min per bed position. Additionally, the subject's age and sex were documented as part of the study. Our imaging protocol was designed following the guidelines set by the Society of Nuclear Medicine and Molecular Imaging [18].

### Quantitative Image Analysis

**Fig. 1:**
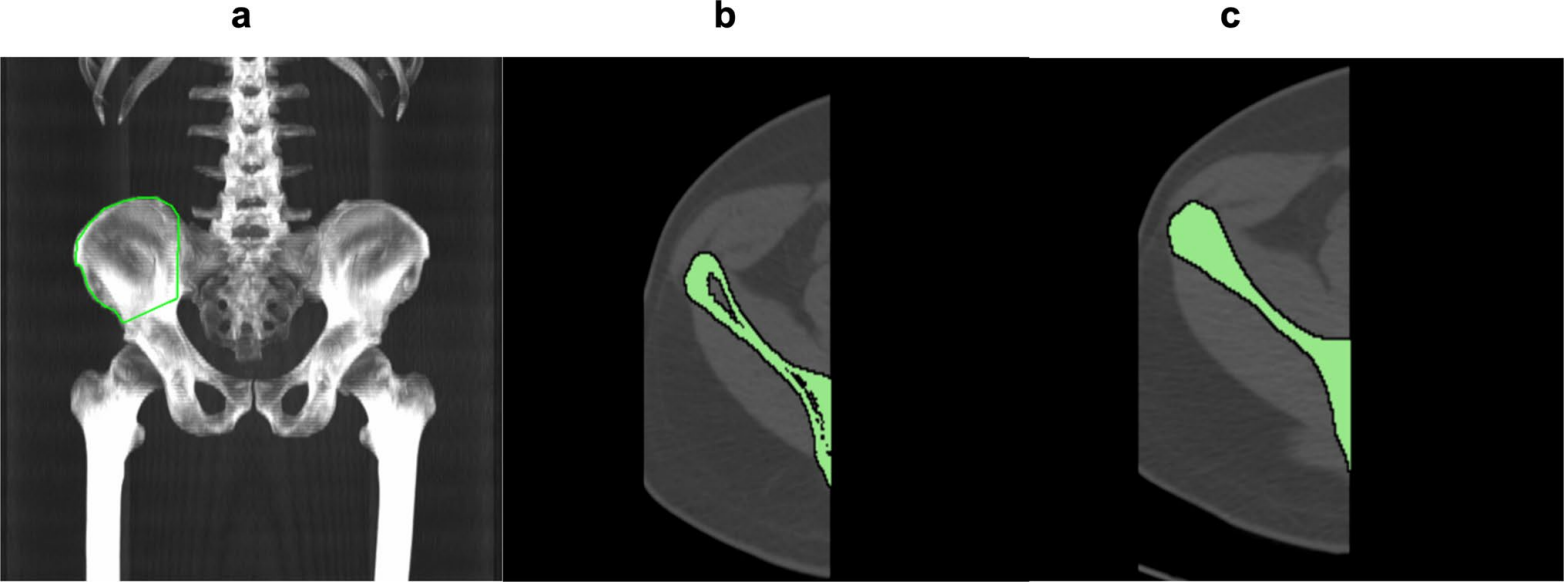
Method of segmentation of iliac bone. (**a**) The region of interest (ROI) is delineated on the iliac bone in 3D MIP. (**b**) After manual anatomical exclusions, a Hounsefield unit-based growing region algorithm is used to segment the cortical portion of the iliac bone. (**c**) The region of interest is then expanded to include the whole medullary cavity using a morphological closing algorithm

OsiriX MD 12.5.1 software (Digital Imaging and Communications in Medicine viewer and image-analysis program; Pixmeo SARL, Bernex, Switzerland) was used for segmentation. A retrospective analysis was done among randomly selected 103 healthy subjects from the CAMONA study. Regions of interest (ROI) were taken on iliac bones in 3D MIP by drawing a straight line joining two points, 2 cm lateral to the upper and lower margin of the sacroiliac joint (SI), the latter being joined to another point through a straight line, 2 cm above the upper margin of the acetabulum (Fig. 1a). The sacroiliac joint (SIJ) and acetabulum were excluded as inflammation within the joints may falsely increase the value. One hundred and three scans were independently analyzed on both sides of the pelvis by generating a fused PET/CT image, and the global mean standardized uptake value (global SUVmean) reflecting [^18^F]NaF uptake was calculated using a Hounsfield unit (HU) threshold-based segmentation algorithm of the OsiriX software version 12.5.1 (Pixmeo, Bernex, Switzerland). Following manual anatomical exclusions, a growing region algorithm was utilized to apply upper and lower thresholds of 1500 Hounsfield units (HU) and 150 HU, respectively, to the combined PET/CT image, segmenting the cortical portion of the iliac bone in the left and right sides of the skeleton separately (Fig. 1b). The ROI was subsequently expanded to include the whole medullary cavity of each bone using a morphological closing algorithm with an element radius of 20 (Fig. 1c). Visual confirmation was made to ensure that the full medullary cavity is included in each patient's ROIs.

### Statistical Analysis

The Shapiro–Wilk test for normality was conducted to assess whether the data followed a normal distribution. Subsequently, a paired t-test was performed to compare [^18^F]NaF uptake between the left and right iliac bones. Pearson correlation coefficient was done to assess the variation in [^18^F]NaF uptake in iliac bones with age, BMI, and physical activity level in both males and females. All statistical tests were performed and the graphs were created using GraphPad Prism version 10.0 (GraphPad Software, Boston, MA, USA).

## Results

The PET findings in this study were obtained from image analysis of healthy subjects with diverse clinical characteristics. This study included 103 healthy subjects from the CAMONA study, 59 males and 44 females, with a mean age of 47.3 ± 13.7 (range: 22–71) years and 47.9 ± 15.9 (range: 21–75) years, respectively. The mean BMI was 27.2 ± 4.46 kg/m^2^. The activity level of the subjects was also recorded, with 18 subjects having a level of 1, 30 subjects having a level of 2, 24 subjects having a level of 3, and 3 subjects having a level of 4. Fifty-five subjects never smoked, 39 sometimes smoked, and nine were active smokers. The mean years smoked and pack-years were 5.9 ± 10.2 (range: 0–34) and 6.1 ± 10 (range: 0–44), respectively. Five subjects never drank, three occasionally drank, and 95 were active drinkers. The mean units per week and alcohol-years were 4.9 ± 5.3 (range: 0–30) and 24.5 ± 14.6 (range: 0–48), respectively. Table 1 summarizes the clinical characteristics of all the subjects included in this study.

**Table 1:** Clinical characteristics of all the study subjects included in this study

Age (in years)	Mean ± SD Range	47.5 ± 14.6 21–75
Gender	Male Female	59 44
BMI	Mean ± SD Range	27.2 ± 4.46 17.8–42.1
Smoking	Never Sometimes Active • Years smoked (Mean ± SD, range) • Pack-years (Mean ± SD, range)	55 39 9 5.9 ± 10.2; 0–34 6.1 ± 10; 0–44
Alcohol	Never Sometimes Active • Units per week (Mean ± SD, range) • Alcohol-years (Mean ± SD, range)	5 3 95 4.9 ± 5.3; 0–30 24.5 ± 14.6; 0–48
Activity level	N/A 1 2 3 4	28 18 30 24 3
Fasting plasma Glucose (mmol/L)	Mean ± SD Range	5.6 ± 0.6 4.4–8.4
History of hypercholesterolemia	Yes No	14 89
History of type II diabetes mellitus	Yes No	0 103
Taking blood pressure lowering medication	Yes No	1 102
Taking diuretics	Yes No	8 95
Taking lipid lowering medication	Yes No	12 89

The right iliac bone had higher [^18^F]NaF uptake compared to the left iliac bone in males, females, as well as when males and females were analyzed together (*P* < 0.0001, in all cases). The average SUVmean of [^18^F]NaF in the iliac bones of males was 2.98 ± 1.63 (range: 1.1—7.87) on the left and 3.71 ± 1.49 (range: 1.49—3.7) on the right side. The average SUVmean of [^18^F]NaF in the iliac bones of females was 2.59 ± 1.14 (range: 0.88—6.27) on the left and 3.72 ± 1.04 (range: 2.22–6.51) on the right side. When both males and females were analyzed together, the average SUVmean of [^18^F]NaF was 2.81 ± 1.44 (range: 0.88—7.87) on the left and 3.71 ± 1.31 (range: 0.89—8.07) on the right side (Table 2andFig. 2).

**Table 2:** Average SUVmean and range of [^18^F]NaF uptake in the left and right iliac bone in male, female, and combined group

Gender	Side/Laterality of the Iliac bone	Average SUVmean	Range
**Male**	Right	3.71 ± 1.49	1.49—3.7
	Left	2.98 ± 1.63	1.1—7.87
**Female**	Right	3.72 ± 1.04	2.22–6.51
	Left	2.59 ± 1.14	0.88—6.27
**Male and female combined together**	Right	3.71 ± 1.31	0.89—8.07
	Left	2.81 ± 1.44	0.88—7.87

**Fig. 2:**
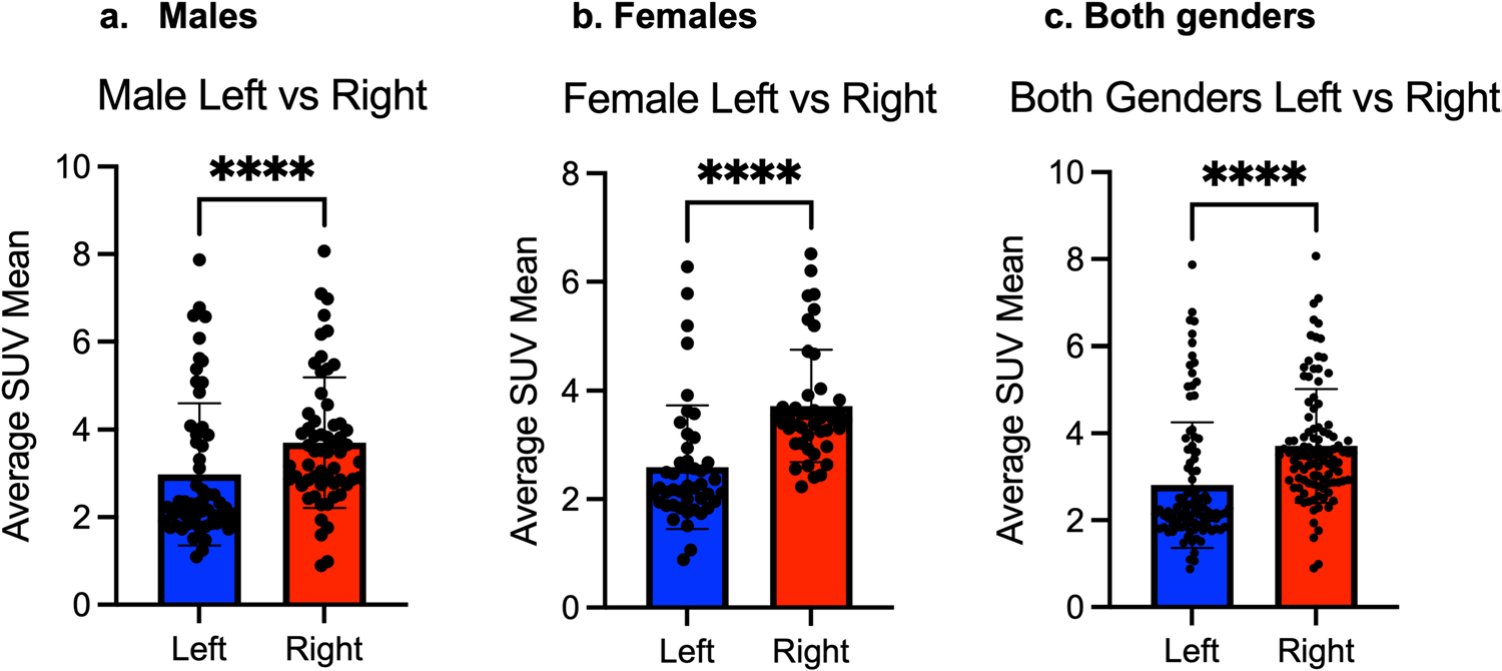
The right iliac bone had higher [^18^F]NaF uptake compared to the left iliac bone in (**a**) males, (**b**) females, and (**c**) when both genders were analyzed together

[^18^F]NaF uptake in the iliac bones displayed a negative correlation with age, evident on both the right (r = − 0.27, *P* = 0.006) and left (r = − 0.22, *P* = 0.02) sides. This negative correlation was more pronounced in females, with Pearson correlation coefficients of − 0.30 (*P* = 0.04) on the right and − 0.31 (*P* = 0.04) on the left side, in contrast to males who exhibited weaker Pearson correlation coefficients of − 0.26 (*P* = 0.04) on the right side and − 0.18 (*P* = 0.18) on the left side. A significant positive correlation was found between the average SUVmean and BMI in males on the right (r = 0.47, *P* = 0.0002) and on the left (r = 0.38, *P* = 0.0027) sides. The correlation was slightly weaker in females on the right (r = 0.36, *P* = 0.0168) and on the left (r = 0.30, *P* = 0.0505) sides. When analyzed together, both genders showed strong correlations on the right (r = 0.43, *P* < 0.0001) side and on the left (r = 0.37, *P* = 0.0001) sides. There was no significant correlation between average SUVmean and physical activity level in males (r = − 0.11 on the right and r = − 0.18 on the left; *P* > 0.05 for both sides). In females, a negative correlation was observed on the left side (r = − 0.37, *P* = 0.0390), although statistically insignificant on the right side (r = − 0.27, *P* = 0.1302). When both genders were analyzed together, a significant negative correlation was found on the left (r = − 0.24, *P* = 0.0372), although statistically insignificant on the right (r = − 0.16, *P* < 0.1541) (Table 3andFig. 3).

**Table 3:** Correlation of age, BMI, and physical activity level with [^18^F]NaF uptake of iliac bones on the right and left sides

Correlations between	Gender	Side/Laterality of the Iliac bone	Pearson correlation coefficient (r)	*P*-value
Age and [^18^F]NaF uptake of Iliac bones	Male	Right	− 0.26	0.042*
		Left	− 0.18	0.181
	Female	Right	− 0.30	0.044*
		Left	− 0.31	0.042*
	Male and female combined together	Right	− 0.27	0.005*
		Left	− 0.22	0.023*
BMI and [^18^F]NaF uptake of Iliac bones	Male	Right	0.47	0.0002*
		Left	0.38	0.0027*
	Female	Right	0.36	0.0168*
		Left	0.30	0.0505
	Male and female combined together	Right	0.43	< 0.0001*
		Left	0.37	0.0001*
Physical activity level and [^18^F]NaF uptake of Iliac bones	Male	Right	− 0.11	0.4768
		Left	− 0.18	0.2420
	Female	Right	− 0.27	0.1302
		Left	− 0.37	0.0390*
	Male and female combined together	Right	− 0.16	0.1541
		Left	− 0.24	0.0372*

**Fig. 3:**
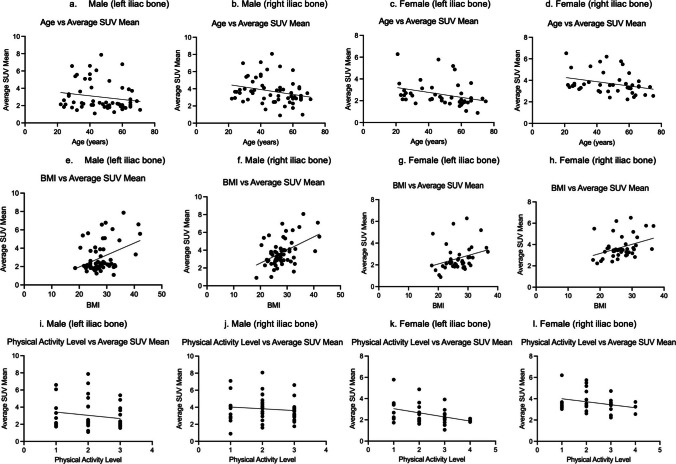
Graphs illustrating the correlations between average SUVmean of [^18^F]NaF and age (**a**–**d**), BMI (**e**–**h**), and physical activity level (**i**–**l**) in males and females for the left and right iliac bones

## Discussion

In this study, we found that [^18^F]NaF uptake was significantly higher in the right iliac bone compared to the left in both males and females and in the combined-gender analysis. Additionally, our findings demonstrated an age-dependent decline in [^18^F]NaF uptake in both genders, suggesting a reduction in bone metabolic activity with aging. We also identified a significant positive correlation between SUVmean and BMI across all groups, indicating a potential link between body composition and bone metabolic activity. A negative correlation was observed between SUVmean and physical activity in the left iliac bone of females and the combined-group, whereas no significant correlation was found in males.

The observed higher [^18^F]NaF uptake in the right iliac bone compared to the left suggests potential functional variations between the two sides. While no studies have specifically investigated laterality in iliac bones, bilateral asymmetry in pelvic bones has been previously reported, arising from both external factors like biomechanical loading and internal factors such as genetics [19]. However, directional asymmetries are generally considered adaptive responses to loading. Mechanical loading plays a critical role in regulating bone mass and morphology by inducing dynamic strains in bone tissue, with peak strain amplitude and strain rate being key predictors of the osteogenic response [20, 21]. Although the pelvis exhibits less directional asymmetry than the limbs, its close association with the lower limbs and role in locomotion suggest that its asymmetries may resemble those found in the lower limbs [22]. Evidence supports right limb dominance in our study cohort, as demonstrated by greater [^18^F]NaF uptake in the right tibia than the left in both male and female subjects in a study by Park et al. [23]. Limb dominance may also be associated with the side of the body where injury occurs [24, 25]. Previous [^18^F]NaF studies assessing bone turnover at the sacroiliac and hip joints suggest that laterality may be associated with increased osteoblastic activity in right-sided weight-bearing bones and joints compared to the left in this cohort [26, 27]. However, the absence of data related to limb preference in the examined subjects limits definitive conclusions for lateral differences in [^18^F]NaF uptake in iliac bone.

Our results revealed an age-dependent decline in iliac bone [^18^F]NaF uptake on PET/CT imaging in both genders, with females showing a stronger negative correlation with age compared to males. This pattern aligns with the pathogenesis and epidemiology of osteoporosis, characterized by an imbalance in bone remodeling where resorption exceeds formation, leading to net bone loss that affects women more than men [28]. Osteoporosis develops through a variety of factors, including genetic predisposition (variants in genes such as vitamin D receptor, collagen type I, and estrogen receptor), hormonal changes (declining levels of estrogen and testosterone), and environmental factors (inadequate calcium intake, vitamin D deficiency, and physical inactivity) [29]. These factors disrupt the tightly regulated bone remodeling process, which involves osteoclastic resorption of old bone followed by osteoblastic formation of new bone [30]. In osteoporosis, excessive osteoclastic activity or inadequate osteoblastic response leads to incomplete refilling of resorption cavities, deteriorating bone mass and microarchitecture [28]. Peak bone mass, typically attained by the third decade of life, significantly influences osteoporosis risk [31], and this maximum bone density depends on genetic factors and modifiable lifestyle elements during development [32]. After reaching peak bone mass, both genders experience age-associated bone loss, but females face accelerated decline. In postmenopausal women, estrogen deficiency upregulates osteoclastogenesis while impairing osteoblastic function [33, 34]. In contrast, males undergo a more gradual decline attributed to decreased testosterone, vitamin D insufficiency, and other comorbidities [35]. Other contributors to excessive bone resorption include glucocorticoid exposure, alcohol consumption, smoking, sedentary lifestyle, and certain metabolic disorders [36]. Our findings align with previous [^18^F]NaF PET/CT studies demonstrating an age-dependent decrease in bone metabolic activity at other skeletal sites typically involved in osteoporosis. Utilizing the same cohort of individuals with or at risk for osteoporosis as previously analyzed by Rhodes et al. in their study of the femoral neck, we applied our novel ROI approach to the iliac bone and observed a similar trend between [^18^F]NaF uptake and age. This suggests that our method yields comparable insights into age-related changes in bone metabolism at a different skeletal site; however, it does not independently validate the findings of Rhodes et al. [16]. Frost et al. found significantly lower vertebral bone plasma clearance values by [^18^F]NaF PET in a cohort of postmenopausal women with osteoporosis compared to osteopenic and normal subjects [17]. In agreement with this existing evidence from established osteoporosis-prone regions like the spine and hip, our data expands the utility of [^18^F]NaF PET/CT to interrogate molecular signatures of aging bone metabolism in the iliac bones.

A significant positive correlation was observed between SUVmean and BMI in males, females, and the combined-group. A weak negative correlation was found between SUVmean and physical activity in the left iliac bone of females and in the combined-group but there was no significant correlation in males. While the relationship between increased BMI and bone formation is relatively well-documented, particularly in the glenohumeral joint [37], the precise underlying mechanisms remain an area of ongoing investigation. Elevated BMI influences bone health through alterations in bone cell metabolism, fluctuations in bone-regulating hormones, and increased oxidative stress and inflammation [38]. Although excessive adipose tissue has been hypothesized to exert a bone-protective effect through mechanical loading and hence stimulating bone formation via increased osteoblast and osteocyte activity, studies that account for overall body weight often fail to support a positive association between fat mass and bone mass [39]. Moreover, excessive fat mass, particularly in individuals with a BMI exceeding 35, has been demonstrated to negatively impact bone mineral density and mechanical strength. Conversely, lean body mass, which is closely associated with physical activity and mechanical loading, has been strongly linked to improved bone mineral density, as muscle activity plays a critical role in promoting bone growth [40]. Therefore, incorporating a detailed analysis of fat mass versus lean body mass on bone formation, and hence [^18^F]NaF uptake, could provide a more comprehensive understanding of these relationships in future studies.

On the other hand, when both genders were analyzed together, a significant negative correlation was observed between mean [^18^F]NaF uptake and physical activity levels. This finding contrasts with established literature, which generally supports a positive relationship between physical activity and bone metabolism, as mechanical loading is known to enhance osteogenesis. However, this discrepancy may be attributed to the specific nature of the physical activity performed, as certain weight-bearing exercises, such as resistance training, are more effective in stimulating osteogenic activity. In contrast, non-weight-bearing exercises do not provide sufficient mechanical loading to promote bone formation. The mechanical load applied during weight-bearing exercises must exceed those experienced during routine daily activities to effectively stimulate new bone formation [41, 42]. Notably, our study did not categorize participants’ physical activity types, and it is plausible that individuals with high physical activity levels predominantly engaged in non-weight-bearing exercises, thereby failing to induce adequate bone formation and resulting in decreased [^18^F]NaF uptake.

To our knowledge, this is the first study evaluating [^18^F]NaF uptake in iliac bones and analyze the variation based on laterality, age, gender, BMI, and physical activity level. This region is an intriguing site for studying systemic metabolic bone disease for several reasons. First, the iliac bones are large, making measurements convenient and potentially reproducible. Second, as part of the pelvic ring, a central weight-bearing structure, the iliac bones are susceptible to fragility fractures, which can lead to significant morbidity and mortality in the aging and osteoporotic population [43]. While the spine and hip are classic sites for bone mineral density measurements during osteoporosis screening, they have limitations. Osteoarthritis can affect these areas, potentially leading to artificially elevated bone mineral density [44]. Additionally, surgeries in the spine and hips are common, making measurements in these sites unreliable due to post-surgical changes [45, 46]. Currently, transiliac bone biopsy sampling of the iliac crest is the only direct method to assess bone remodeling dynamics at the tissue level through quantitative histomorphometric analysis [14]. Previous histomorphometric studies on iliac crest biopsy samples have shown age-related microstructural deterioration of trabecular bone, including decreased trabecular thickness, increased trabecular separation, compromised bone formation rates, and elevated erosion surfaces. These findings are consistent with the profile of exaggerated bone turnover and remodeling imbalance characterizing osteoporosis [14, 47]. However, these invasive transiliac procedures are limited by sampling errors and are rarely performed outside of research settings due to their invasive nature. These limitations highlight the significant implications of our study. [^18^F]NaF PET/CT imaging of iliac bones could serve as a non-invasive alternative for assessing bone metabolism, offering a new modality for how we diagnose, monitor, and treat metabolic bone diseases like osteoporosis in both research and clinical settings.

Our retrospective analysis of an otherwise healthy cohort from the CAMONA study had several strengths, including comprehensive clinical phenotyping and quantitative assessment of [^18^F]NaF uptake within the iliac bones on a per-subject basis. However, the study lacked correlative measurements of bone mineral density, clinical history of fractures, and other potential risk factors for metabolic bone disease. Future studies should incorporate this data to relate [^18^F]NaF uptake patterns to clinically relevant osteoporosis outcomes and assess diagnostic performance against gold-standard measures of skeletal fragility. Additionally, establishing age-, sex-, and BMI-matched reference ranges for quantitative [^18^F]NaF uptake parameters in the iliac bones through larger normative databases will be necessary to enhance diagnostic performance for identifying individuals with metabolic bone disease. Exploring the influence of factors like physical activity levels on iliac bone metabolism and conducting longitudinal evaluations to track changes in iliac [^18^F]NaF uptake over time will further clarify the relationship between molecular bone activity and structural deficits that increase fracture risk, ultimately defining imaging biomarker thresholds for initiating clinical interventions.

## Conclusions

Our study identified the potential of [^18^F]NaF PET/CT for assessing iliac bone metabolism in healthy subjects from CAMONA study. The right iliac bone had higher [^18^F]NaF uptake compared to the left iliac bone, representing the effects of laterality on iliac bone turnover. The observed associations of lower iliac bone [^18^F]NaF uptake with older age, more pronounced in females, are consistent with established concepts of accelerated bone loss and turnover imbalance that predispose to osteoporosis. A positive correlation between BMI and SUVmean reinforces the concept that greater mechanical loading leads to increased bone remodeling. In contrast, a weak negative correlation between physical activity level and SUVmean for the left iliac bone in females and combined-group suggests a possible differential effect of weight-bearing and resistant exercises vs other types of exercises on bone metabolism. Further research is necessary to validate these findings in a larger cohort, establish quantitative reference scales, and explore relationships with clinical outcomes to improve fracture risk assessment and therapeutic monitoring, possibly beyond what conventional bone mineral density measurements offer at this time.

## Data Availability

Anonymized research data are available from the corresponding author upon request.
